# A prospective cohort study of shock index as a reliable marker to predict the patient’s need for blood transfusion due to postpartum hemorrhage

**DOI:** 10.12669/pjms.37.3.3444

**Published:** 2021

**Authors:** Suleyman Cemil Oglak, Mehmet Obut, Ali Emre Tahaoglu, Neslihan Ugur Demirel, Bekir Kahveci, Ihsan Bagli

**Affiliations:** 1 Suleyman Cemil Oglak, Department of Obstetrics and Gynecology, Health Sciences University, Gazi Yasargil Training and Research Hospital, Diyarbakir, Turkey; 2 Mehmet Obut, Department of Obstetrics and Gynecology, Etlik Zubeyde Hanım Women’s Health Training and Research Hospital, Ankara, Turkey; 3 Ali Emre Tahaoglu, Department of Obstetrics and Gynecology, Memorial Dicle Hospital, Diyarbakır, Turkey; 4 Neslihan Ugur Demirel, Department of Obstetrics and Gynecology, Health Sciences University, Gazi Yasargil Training and Research Hospital, Diyarbakir, Turkey; 5 Bekir Kahveci, Department of Obstetrics and Gynecology, Cukurova University School of Medicine, Adana, Turkey; 6 Ihsan Bagli, Department of Obstetrics and Gynecology, Health Sciences University, Gazi Yasargil Training and Research Hospital, Diyarbakir, Turkey

**Keywords:** Postpartum hemorrhage, Shock index, Vaginal delivery

## Abstract

**Objective:**

This study was aimed to compare the shock index (SI) values between patients who required blood transfusion due to postpartum hemorrhage (PPH) and patients who received no blood transfusion.

**Methods:**

We conducted this cross-sectional study at a tertiary center between January 2019 and June 2019. A total of 2534 patients who underwent vaginal delivery were included in this study. We measured SI values upon admission, 30 minutes, 1-hour, and 2-hours after delivery. We identified women who required blood transfusion as the study group. Control patients who delivered in the same period and received no blood transfusion were identified in the medical record system and randomly selected. Age, parity, BMI, and SI values at each one prepartum and three postpartum periods of the groups were analyzed.

**Results:**

A total of 2534 patients were included in the study. A varying amount of blood transfusion was performed in 54 patients (2.13%). When we compared with patients who did not receive blood transfusion after delivery, patients who received any amount of blood transfusion after vaginal delivery had significantly higher SI values 30 minutes after delivery (0.99±0.20, and 085±0.11, p=0.0001), at 1-hour (1.00±0.18, and 0.85±0.11, p=0.0001), and 2-hours (1.09±0.16, and 0.87±0.11, p=0.0001).

**Conclusion:**

SI value could be a reliable and consistent marker to predict the requirement for any amount of blood transfusion due to PPH.

## INTRODUCTION

Postpartum hemorrhage (PPH) remains one of the major causes of maternal death, accounting for 27.1% of all maternal deaths worldwide in the third trimester of pregnancy.^1^,^2^ PPH is defined as the total blood loss ≥1000 ml within 24 hours after the delivery process (includes intrapartum loss) regardless of route of delivery.^3^ The prevalence of PPH varies between 1-10% of all deliveries.^4^ Overall, 17.2% of PPH patients results in maternal near-miss or maternal death, and the rates of severe maternal outcomes (SMO) are higher in low- and middle-income countries.^5^ The main point is that early recognition and prompt intervention are crucial to reducing hemorrhage-related SMO.^6^,^7^ Studies have showed that visual estimation is more likely to underestimate the exact blood loss when volumes are high and overestimate when blood loss is low. However, there is no clinical evidence of the effectiveness of quantitative blood loss measurement on maternal outcomes.^8^

The conventional vital signs, including systolic blood pressure (SBP) and heart rate (HR), are used most commonly by clinicians for determining hemodynamic stability, identifying patients in emergency medical circumstances, and triggering an escalation of maternal care.^9^ These signs have poor predictive value in pregnant patients due to both the hemodynamic changes of pregnancy and compensatory physiological responses of early hemorrhagic shock.^10^ Changes in vital signs occur lately that the patient is already in a risky condition, leading to delays in the essential intervention.^11^ Therefore, imminent hypovolemic shock may be hidden by the hemodynamic changes of gestation, causing conventional vital signs less helpful, and signs taken in isolation may neglect impending worsening.^6^

Clinicians emphasize a need for early warning criteria for PPH, which has a higher sensitivity to physiologic changes, ease of application in clinical practice, and prioritization of both the lost blood volume and its clinical outcomes, to indicate the severity of blood loss.^12^ Shock index (SI), the ratio of HR divided by SBP, has been proposed as an early and reliable predictor of adverse outcomes in non-obstetric trauma and obstetric hemorrhage patients compared with conventional vital signs.^6^ Clinical and experimental studies have shown that SI has an inverse linear correlation with left ventricular stroke work in acute circulatory failure. Hence, a reduction of left ventricular stroke work due to trauma, hemorrhage, or sepsis was associated with an increase of the SI and a worsening in left ventricular mechanical performance.^13^ The normal range for SI in non-obstetric patients has been considered to be 0.5 to 0.7.^10^ However, most of the studies have proposed the upper limit of SI for PPH patients as 0.9 in both low- and well-resourced facilities to predict adverse outcomes.^6^,^14^ The current study was aimed to compare the SI values between patients who required blood transfusion due to PPH and patients who received no blood transfusion.

## METHODS

We conducted this cross-sectional study at Diyarbakir Gazi Yasargil Training and Research Hospital between January 2019 and June 2019. A total of 2534 patients who underwent vaginal delivery were included in this study. All patients were above 34 weeks of gestation and delivered within the borders of the hospital. We obtained informed consent from all participants. The Ethics Committee of the same hospital approved the study (Ref # 199, dated December 28, 2018).

Patients with gestational hypertensive disorders, infections with fever, sepsis, cardiac diseases, hypo- or hyperthyroidism were excluded. Patients with previously used antihypertensive treatment, received a blood transfusion during the antenatal period, delivered by cesarean section, or experienced surgical interventions due to severe bleeding were also excluded. Epidural anesthesia was not utilized for any patient for pain management during the labor progress. We extracted data from the patients’ medical records. Patients with missing data or those who no longer wanted to participate were excluded.

The demographic characteristics of all patients, including age, parity, and body mass index (BMI), were recorded. Accordingly, to our clinical protocol, we performed active management in the third stage of all vaginal deliveries.^15^ A drape was located under each patient’s hip promptly after all vaginal deliveries to estimate the blood loss. The volume of blood loss was assessed by visual examination of the drapes during the follow-up period. The threshold estimated blood volume loss for PPH was ≥1000 ml after the vaginal delivery. All patients experienced evidence-based hemorrhage management, if required.^16^ We measured vital sign parameters, including HR, SBP, and SI, for application as early warning criteria. HR and SBP were measured by an electronic cuff connected with an automatic monitor, and these results were recorded. The SI was calculated by dividing the HR by SBP. We evaluated these vital signs upon facility admission, 30 minutes, 1-hour, and 2-hours after delivery.

We identified women who required blood transfusion as the study group. Control patients who delivered in the same period and received no blood transfusion were identified in the medical record system. Age, parity, BMI, and SI values at each one prepartum and three postpartum periods of the groups were analyzed.

### Statistical analysis:

The sample size was calculated using the G-Power version 3.1.9.4 (Universitat Kiel, Germany), regarding the values indicated in the previous studies.^10^ The minimum number of patients to be included in the study was 82 (41 PPH patients and 41 controls), with a two-tailed alpha error of 5% and a power of 91%. Since 2534 patients (54 severe PPH cases and 2480 controls) were included in our study, the power of the study was calculated as 100%.

In this study, statistical analysis were performed with Number Cruncher Statistical System (NCSS) 2007 Statistical Software (Utah, USA) package program. Measured variables were presented as mean±standard deviation (std), and categorical variables were presented as numbers and percentages (%). An independent t-test was used for comparison of binary groups, and the chi-square test was used for comparison of qualitative data. The results were evaluated at the significance level of p<0.05.

## RESULTS

During the study period, a total of 3541 deliveries took place. Sixty-two of these patients received a blood transfusion due to anemia during the antepartum period, 252 of them had gestational hypertensive disorders, and all of them were excluded from the study. Thirty-six patients experienced instrumental delivery and were excluded from the study. Also, 657 patients were excluded from the study due to delivery by cesarean section or other exclusion criteria. A total of 2534 patients were included in the study.

The demographic characteristics, and blood transfusion rates of the patients were summarized in Table-I. The mean age of the patients was 27.28±5.95, and the mean BMI of the patients was 24.89±4.87 kg/m^2^. A varying amount of blood transfusion was performed in 54 patients (2.13%). Forty of these patients received one unit, 12 of them received two units, and two of them received three units of red blood cell transfusion.

**Table-I:** Demographic characteristics and blood transfusion rates of the patients.

		N	%
Age	<19	218	8.60
	20-24	696	27.47
	25-29	784	30.94
	30-35	496	19.57
	>35	340	13.42
Parity	Nulliparous	308	12.15
	1-4 Multiparous	1990	78.53
	>5 Grand Multiparous	236	9.31
BMI	<18	248	9.79
	19-24	1098	43.33
	25-29	762	30.07
	30-34	426	16.81
Blood transfusion	No	2480	97.87
	Yes	54	2.13

The relationship between the demographic characteristics and blood transfusion rates of the patients is summarized in Table-II. Blood transfusion requirements were significantly higher in nulliparous patients and significantly lower in multiparous patients (p=0.037, and p=0.008, respectively).

**Table-II:** The relationship between the demographic characteristics and blood transfusion rates of the patients.

		Blood transfusion (-)		Blood transfusion (+)		p
Age	<19	210	8.47%	8	14.81%	0.164
	20-24	682	27.50%	14	25.93%	0.918
	25-29	770	31.05%	14	25.93%	0.562
	30-35	484	19.52%	12	22.22%	0.747
	>35	334	13.47%	6	11.11%	0.763
Parity	Nulliparous	296	11.94%	12	22.22%	0.037
	1-4 Multiparous	1.956	78.87%	34	62.96%	0.008
	>5 Grand multiparous	228	9.19%	8	14.81%	0.242
BMI	<18 BMI	240	9.68%	8	14.81%	0.305
	19-24 BMI	1.079	43.51%	19	35.19%	0.279
	25-29 BMI	742	29.92%	20	37.04%	0.327
	30-34 BMI	419	16.90%	7	12.96%	0.561

Mean SI values in patients with and without blood transfusions are summarized in Table-III. The mean SI values in the patients who underwent blood transfusions were significantly higher in the postpartum 30 minutes, 1-hour, and 2-hours (p=0.0001).

**Table-III:** Mean SI values in patients with and without blood transfusions

	Blood transfusion (-) n: 2480	Blood transfusion (+) n: 54	p
Prepartum SI	0.76±0.07	0.77±0.08	0.110
Postpartum 30 min SI	0.85±0.11	0.99±0.20	0.0001
Postpartum 1-hour SI	0.85±0.11	1.00±0.18	0.0001
Postpartum 2-hours SI	0.87±0.11	1.09±0.16	0.0001

## DISCUSSION

In this study, we aimed to clarify the role of SI in the identification of blood transfusion requirements due to severe PPH and to develop early warning criteria that could assist in PPH patient’s identification. We found that SI values after vaginal delivery can identify patients with severe PPH. The SI values were significantly higher in patients who required blood transfusion for severe bleeding than among patients who did not receive it.

Maternal blood volume increases by early weeks of gestation and peaks by 32-34 weeks with an increase of 40-50%. The significant volume expansion may protect the patients from adverse outcomes due to bleeding during pregnancy and immediately after delivery.^17^ Therefore, a healthy pregnant and postpartum patient can lose up to 30% (approximately 1500 mL) of her blood volume without any significant change in conventional vital signs. These compensatory mechanisms can hide hypovolemia, cause a presupposition of hemodynamic stability, and delay in maternal care.^18^

SI has been demonstrated to be consistently an early warning criterion to predict the severe PPH and trigger the escalation of care^5^. Previous studies have suggested different thresholds of SI values in the postpartum period to alert healthcare providers early recognition and more rapid intensive treatment. Nevertheless, all studies have found that the increase of SI value in the postpartum period is associated with the risk of blood transfusion, surgical intervention, and severe maternal outcomes.^12^,^19^ Most of the studies have reported that SI values ≥0.9 are associated with a blood transfusion of ≥4 units and severe maternal outcomes due to PPH.^6^,^18^ This value is greater than the non-obstetric patients where the upper limit of normal is 0.7. This difference can be explained by the hemodynamic changes that occur during pregnancy and labor, in that a rise in resting HR, which often increase even more in the immediate postpartum period due to pain and effort.^20^

In a study by Le Bas et al., the mean SI at 10 and 30 minutes was 0.91 and 0.90, respectively, with 64% requiring blood transfusion.^21^ In the same study, 89% of patients with an SI of ≥1.1 at 10 minutes and 75% with an SI of ≥1.1 at 30 minutes required blood transfusion. They proposed that an SI of ≥1 (HR is greater than or equal to SBP) may be a useful adjunct in estimating the blood loss and in predicting the need for blood transfusion. In the study of Borovac-Pinheiro et al., the mean SI value among patients who experienced any amount of blood transfusion after vaginal delivery due to PPH was 0.88±0.26 at 30 minutes, and 0.90±0.23 at 2-hours.^12^ In this study, when we compared with patients who did not receive blood transfusion after delivery, patients who received any amount of blood transfusion after vaginal delivery had significantly higher SI values 30 minutes after delivery (0.99±0.20, and 085±0.11, p=0.0001), at 1-hour (1.00±0.18, and 0.85±0.11, p=0.0001), and 2-hours (1.09±0.16, and 0.87±0.11, p=0.0001). We investigated a large number of patients with a diversity of obstetric hemorrhage etiologies, and prioritize experiencing any amount of blood transfusion due to PPH. We found that elevated SI value was related to the requirement for blood transfusion, and we consider that SI implicates the hemodynamic instability, and is a reliable marker for the prediction of severe PPH. SI provides prompt management, including preparation of the blood products for transfusion to carry out to reduce hypovolemic shock.

WHO recommends the visual estimation of blood loss for assessing the severity of vaginal bleeding.^4^ However, studies have reported that visual estimation is a 30-50% underestimation of blood loss.^22^ In patients with anemia, the upper limit of SI will increase due to the tachycardic response. Also, since less amount of blood loss can cause hemodynamic instability, SI displays a more useful assessment tool than estimated blood loss. Hemoglobin value does not reflect the severity of blood loss in the early period of PPH, and hemoglobin value is affected by the therapeutic approaches, including intravenous fluids and blood transfusion.^18^ Therefore, SI value is more suitable for predicting the blood transfusion in women with PPH.

Most of the amount of blood loss occurs within two hours after delivery.^23^ Borovac-Pinheiro et al. stated that PPH treatment should be started as suspected to prevent excessive blood loss. This initiation should be within the first hour of delivery, preferably within 30 minutes of delivery.^12^ Therefore, in the current study, we followed-up the vital signs during the first two hours of delivery to diagnose severe PPH early to enable prompt handling.

Theoretically, iatrogenic approaches could impact HR, SBP, and so SI value. In our hospital setting, we performed active management in the third stage of all vaginal deliveries with uterotonic administration (oxytocin and/or ergometrine) to the patient. Nathan et al. reported that the use of oxytocin and ergometrine had negligible effects on the SI value in PPH patients.^18^ All transfused patients received intravenous hydration before blood transfusion. Some of the patients experienced perineal trauma repair due to the laceration of the perineum. This procedure can cause pain immediate postpartum period, which can increase the patient’s SI value. However, in our study, no patient received a pain killer due to the complaint of severe pain.

We excluded several participants who were massively bleeding, therefore experiencing surgical interventions, and receiving massive blood transfusions during continuing blood loss. We consider that performing a blood transfusion to the patient concurrent with bleeding reduces the accuracy of the SI value that should occur as a physiological response. We also excluded all patients with gestational hypertensive disorders since the SI value is calculated by dividing HR to SBP. Although Kohn et al. reported that peak SI values did not change in patients with gestational hypertension, we consider that these patients should be uniquely evaluated in accordance with the hemodynamics of gestational hypertension. We excluded emergency cesarean section patients and patients who underwent major surgical intervention immediately after delivery. Nathan et al. reported that in such cases, the predictive value of SI was less.^18^

### Limitations of the study:

We performed a visual estimation of blood loss with noncalibrated drapes and did not quantify with blood-soaked items. Therefore, the amount of blood loss may have been evaluated with lower accuracy. As our clinic is a maternity unit in a tertiary referral center, patients are closely followed-up, and intravenous fluid treatment is started as one of the initial resuscitation steps of bleeding. Patients were not classified according to the amount of intravenous fluid they received. This treatment may have affected the SI value.

### Strengths of the study:

The strength of this study is that our SI values represent a four-time point as one of them is in the intrapartum and three of them are within the two hours of the postpartum period. Therefore, SI value utilized more accurately to determine the blood transfusion requirement. Also, all patients with PPH managed with the same protocol before receiving the blood transfusion. HR and SBP of the patients measured with automated devices to minimize the user fault and improve the accuracy of the measurement.

## CONCLUSION

This study has showed that SI value could be a reliable and consistent marker to predict the requirement for any amount of blood transfusion due to PPH.

### Author’s Contribution:

**SCO, MO, AET and IB:** Responsible and accountable for this study, conception and design of the study, data collection and processing, analysis and interpretation of data, literature review.

**SCO:** Writing the manuscript, critical review.

**NUD and BK:** Conception and design of the study, data collection and processing, analysis and interpretation of data, literature review.
